# Prevalence of Tobacco Smoking and Associated Risk Factors Among Public Sector Employees in Kuwait: A Cross-Sectional Study

**DOI:** 10.7759/cureus.35925

**Published:** 2023-03-09

**Authors:** Diaa Soliman, Sahad Al Akram, Abdulmuhsen AlMutairi, Khalaf AlShammari, Manar Al Hubaidah, Mohammed AlMaayoufi, Moudhi AlMutairi, Sarah AlDaihani, Shaimaa AlKhudher, Ahmed F Alkandari

**Affiliations:** 1 Community Medicine, Kuwait University, Hawally, KWT; 2 General Surgery, Jaber Al-Ahmad Al-Sabah Hospital, South Surra, KWT; 3 Internal Medicine, Sabah Hospital, Shuwaikh, KWT; 4 Pediatrics, Kuwait University, Hawally, KWT; 5 Epidemiology and Public Health, Kuwait University, Hawally, KWT; 6 Anatomy, Kuwait University, Jabriya, KWT; 7 Surgery, Al-Adan Hospital, Al-Ahmadi, KWT

**Keywords:** factors, public health, smoking, shisha, e-cigarettes, cigarettes, tobacco use, prevalence

## Abstract

Introduction

There needs to be more evidence about the tobacco products utilized by individuals who smoke and the primary determinants contributing to the development of smoking behavior. Our study aimed to assess the prevalence and factors associated with using one or more tobacco products among employees from various ministries in Kuwait.

Methods

A cross-sectional study was conducted among employees in different ministries in Kuwait from December 27, 2018, to January 3, 2019. A questionnaire about smoking status and socio-demographic variables was used.

Results

There was a total of 1057 participants in this study. Of the participants, 26% (n=275) reported using at least one tobacco product. The proportion of smoking of at least one tobacco product was higher among men (n=243, 46.5%) than women (n=32, 6%). Among smokers, 1.5%, 5.9%, and 18.6% reportedly use only three, two, and one tobacco products, respectively. Of the study participants, 26% were smokers, 20.3% were exclusively cigarette smokers, and 21.8% reportedly started cigarette smoking at the age of 15 years or less. Male compared to female workers had higher odds of being smokers of at least one tobacco product (adjusted OR= 15.3, 95% CI= 10.0-23.4). Participants were significantly (*p*= 0.009) more likely to use at least one tobacco product if their monthly income in Kuwaiti Dinars ranged from 501-1000 KD (adjusted OR= 1.9, 95% CI= 1.2-3.0) or 1501-2000 KD (adjusted OR= 2.3, 95% CI= 1.2-4.5) compared to those who had monthly income range 500 KD or less.

Conclusion

The male gender and high income of the participants were significant predictors of the use of at least one tobacco product. Anti-smoking campaigns, mass media interventions, and increasing tobacco product taxes may minimize this population's tobacco consumption.

## Introduction

Tobacco is the most important avoidable risk factor for non-communicable diseases. Annually, almost 6 million people worldwide die because of diseases attributed to tobacco use, and this number is expected to increase to 7.5 million by 2020, accounting for 10% of all deaths. In 2015, smoking caused more than one in ten deaths worldwide, killing more than 6 million people with a global loss of nearly 150 million disability-adjusted life-years [1]. Lung, oral, and nasopharyngeal cancers are some of the significant tobacco consumption-related cancers. Unless immediate steps are taken to reduce smoking rates, the number of deaths due to tobacco use is expected to rise to 10 million per year over the next 30-40 years, and 70% of these deaths are likely to occur in developing countries [2].

Nicotine is an active ingredient of tobacco smoke, affecting multiple body organ systems. In the central nervous system, nicotine reaches the brain within a few seconds of inhalation and changes one's mood. Smoking increases the atrophy of the optic nerve, which impairs vision [3]. In the respiratory system, inhaling nicotine leads to the inability of the lungs to filter out toxic chemicals and renders smokers more susceptible to respiratory infections [4]. Additionally, tobacco smoking increases the blood's low-density lipoprotein (LDL) level and reduces the high-density lipoprotein (HDL). This eventually leads to the accumulation of fatty substances in arteries and causes atherosclerosis, which increases the risk of blood clots, recurrent coronary heart disease, and heart attacks [5]. Furthermore, inhaling different kinds of tobacco products reduces one's appetite, making the affected person unable to get all the needed nutrients for good health. Consequently, tobacco use by a large proportion of a country adds a burden to the national economy by increasing costs in health expenditure and other indirect costs due to tobacco smoking-related diseases. 

In the Middle East and Africa, cigarette consumption increased by 57% between 1990 and 2009 [6]. Furthermore, it is estimated that the prevalence of smoking among men is nearly ten times higher than that of women worldwide [7]. The prevention and treatment of tobacco addiction have been targeted by the World Health Organization (WHO) as the priorities for intervention in developing countries. Recently, Global Framework Convention on Tobacco Control set forth the goal to reduce tobacco use prevalence by 30% by 2025 [8].

There is limited evidence about the range of tobacco products adults use and the leading factors to smoking habit in the Middle East, including Kuwait use. Cigarettes, cigars, pipe, and shisha are some of the most common modalities used in indulging in tobacco products used in Kuwait, with more females (69%) smoking shisha compared to males (57%) [9]. Another relatively recent study on tobacco use among male industrial workers in Kuwait reported a prevalence of 34.8% [10]. However, little is known about smoking behavior patterns, including various tobacco consumption modalities in Kuwait.

Understanding the current tobacco smoking burden, including the prevalence of smoking and tobacco consumption through different smoking modalities among adults in Kuwait, may provide scientific evidence for effectively controlling and preventing tobacco-related diseases. Therefore, this study sought to i) assess the prevalence of tobacco smoking and concurrent use of multiple tobacco products in public sector workers and ii) examine the factors associated with the smoking status in the study population. 

## Materials and methods

Ethical approval

Health Sciences Center Ethics Committee approved the study protocol and instrument for Students' Research under the Institutional Review Board (IRB reference number: 4051/2018). As noted above, written informed consent was obtained from all the participants. The participants were assured about the complete confidentiality of their personal information and the anonymity of their responses. It was explained to them that their participation was voluntary and they could withdraw from the study at any point.

Study design, setting, and participants 

A cross-sectional study was conducted among workers in different ministries of the State of Kuwait from December 27, 2018, to January 3, 2019. The sampling frame comprised individuals of either gender, 21 years old or older, and employed full-time in one of the selected ministries, including the Ministry of Commerce and Industry, Ministry of Defense, Ministry of Foreign Affairs, Ministry of Interior, Ministry of Education, and Ministry of Health.

Questionnaire validity and reliability

A self-administered questionnaire comprising 39 items was used to collect data (Table 1). The items were grouped into sections of socio-demographic characteristics and tobacco smoking practices, and comorbidities. Items were questions designed around study objectives based on the literature reviewed and previous similar questionnaires. Two faculty members of the Department of Community Medicine and the Faculty of Medicine at Kuwait University reviewed the questionnaire. Each participant took, on average, approximately 7-8 minutes to complete the questionnaire.

**Table 1:** The study questionnaire that is distributed to the participants.

Study Questionnaire
Q1. What is your gender?
1 Male
2 Female
Q2. What is your age? ________ years
Q3. What is your nationality?
1 Kuwaiti
2 Non-Kuwaiti Arab
3 Non-Kuwaiti Non-Arab
Q4.What governorate do you live in?
1 Al-Ahmadi
2 Capital
3 Al-Farwaniyah
4 Hawalli
5 Al-Jahrah
6 Mubarak Al-Kabeer
Q5. What is your marital status?
1 Single
2 Married
3 Divorced
4 Widow
Q6. What ministry do you work at?___________
Q7. How many years do you work at this ministry?___________
Q8. My work is ___________ .
1 In the morning
2 In the evening
3 Shift based
Q9. How many hours do you work per week?
1 30 hours or less
2 30 – 35
3 35 – 40
4 40 – 45
5 45 hours or more
Q10. I describe my work as stressful.
1 Strongly Disagree
2 Disagree
3 Disagree
4 Neutral
5 Agree
Q11. I believe my sleep is disturbed by my work.
1 Strongly Disagree
2 Disagree
3 Disagree
4 Neutral
5 Agree
Q12. I describe my home as stressful.
1 Strongly Disagree
2 Disagree
3 Disagree
4 Neutral
5 Agree
Q13. What is your total monthly household income? (In K.D.)
1 500 K.D. or less
2 500-1000
3 1000-1500
4 1500-2000
5 2000 K.D. or more
Do you currently have any of the following doctor-diagnosed conditions?
Q14. Heart disease
No
Yes
Q15. Hypertension (high blood pressure)
No
Yes
Q16. Diabetes
No
Yes
Q17. Overweight/obesity
No
Yes
Q18. Frequent upper respiratory tract infections(more often than once a month)
No
Yes
Q19. Peptic ulcer disease or other digestive problems
No
Yes
Q20. Anxiety
No
Yes
Q21. Depression
No
Yes
Q22. Sleep disturbances
No
Yes
Q23. Are you currently a cigarette smoker?
No
Yes
If No, Go to Q26. If Yes, continue to Q24.
Q24. At what age did you start smoking cigarettes? (years)
Q25. How many packs do you smoke per day?
1 Less than one pack
2 One pack
3 Two packs
4 More than two packs
Q26. Are you currently a shisha smoker?
No
Yes
If No, Go to Q29. If Yes, continue to Q27.
Q27. At what age did you start smoking shisha? (years)
Q28. How often do you smoke shisha a week?
1 Once
2 Twice
3 Three times
4 More than three times
Q29. Are you currently an e-cigarette smoker?
No
Yes
If No, Go to Q32. If Yes, continue to Q30.
Q30. At what age did you start smoking e-cigarettes? (years)
Q31. How many vapes do you smoke per day?
1 Less than one vape
2 One vape
3 Two vapes
4 More than two vapes
Q32. Does any member of your family smoke?
No
Yes
If No, Go to Q35. If Yes, continue to Q33.
Q33. who smokes in your family?
1 Grandfathers
2 Grandmothers
3 Father
4 Mother
5 Brothers
6 Sisters
Q34. What does he/she smoke?
1 Cigarettes only
2 Shisha only
3 E-cigarettes only
4 Cigarettes & shisha
5 Cigarettes & e-cigarettes
6 Shisha & e-cigarettes
7 All of them
Q35. Does any of your friends smoke?
No
Yes
If Yes, Go to Q36.
Q36. What does he/she smoke?
1 Cigarettes only
2 Shisha only
3 E-cigarettes only
4 Cigarettes & shisha
5 Cigarettes & e-cigarettes
6 Shisha & e-cigarettes
7 All of them
8 Differs from one friend to another
(If you smoke any tobacco products please answer questions 37&38.)
Q37. How much do you spend each month on smoking products? ( in K.D.)
1 < 25
2 25 – 50
3 50 – 75
4 > 75
Q38. What is the most important factor that is associated with your smoking habit?
1 Stress
2 Peer pressure
3 Trying new things
4 Other ______________ (Mention it.)
(If you currently smoke more than one tobacco product please answer the following question.)
Q39. What is the order of the beginning of your smoking habits?
1 Cigarettes then Shisha
2 Shisha then Cigarettes
3 Cigarettes then E-Cigarettes
4 E-Cigarettes then Cigarettes
5 Shisha then E-Cigarettes
6 E-Cigarettes then Shisha
7 Cigarettes then Shisha then E-Cigarettes
8 Cigarettes then E-Cigarettes then Shisha
9 Shisha then Cigarettes then E-Cigarettes
10 Shisha then E-Cigarettes then Cigarettes
11 E-Cigarettes then Cigarettes then Shisha
12 E-Cigarettes then Shisha then Cigarettes
Thank you for your participation.

Measures

Socio-demographic variables: Questions on self-reported age, gender, nationality, and income were included. 

Instruments for Tobacco use: Questions to assess the consumption of different tobacco products, including smoking onset and the amount consumed daily. Questions to assess the shift between using different tobacco products were also included, e.g., "What is the order of your smoking habits since the beginning?".

Comorbidities: Questions about self-reported or physician-diagnosed tobacco-related morbidities were also included.

Sampling, sample size, and data collection 

The data were collected from December 27, 2018, to January 03, 2019. A proportionate random sampling technique was used to recruit participants in the study. There was a total of 15 ministries in Kuwait with over 160,000 employees. A systemic random sampling was used to choose eight ministries. The total number of employees in those chosen ministries was around 60,000 employees. In order to calculate the sample size, Slovin's formula was used n = N/(1 + N e2), where n = number of samples, N = Total population, and e = margin of error, (0.03), which resulted in an n = 1090. Four data collection teams, comprising two medical students (one with three members), were constituted to accomplish the fieldwork. Workers were approached during their break periods. The objectives of our study were explained to them, and they were invited to participate. Consenting workers were requested to fill out the questionnaire and sign the consent form attached at the front of the questionnaire. Completed questionnaires were checked immediately to spot any oversight by the workers.

Data Analysis

Descriptive statistics, including proportions (%), means, standard deviations, medians, and interquartile ranges, were computed as required to examine the distribution study variables, i.e., demographics, smoking practices, and comorbidities. Chi-square analysis was used to test for the statistical significance (p≤ 0.05) of the association between smoking status and socio-demographics and comorbidities. Multivariate logistic regression analysis was also used to identify the variables independently and significantly (p< 0.05) related to smoking status. The estimated adjusted odd ratio (OR) and their 95% confidence interval (CI) were used to interpret the final multivariate logistic regression model. Data were managed and analyzed using the SPSS statistical analysis software (version 25).

## Results

Description of the study sample

**Figure 1:**
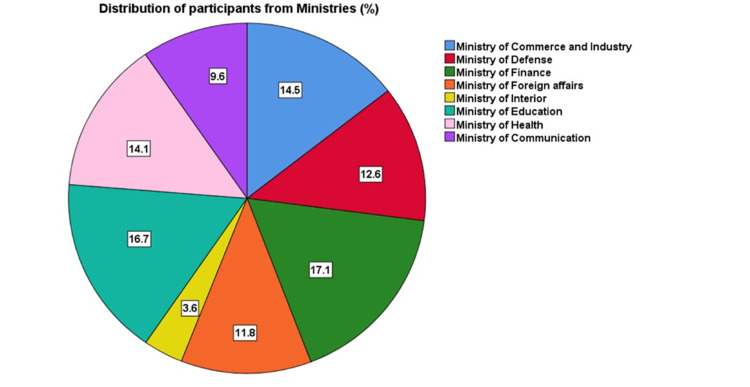
Distribution of the sample of the employees enrolled in the study

A total of 1057 workers from different ministries of Kuwait participated in the current study, of whom 523 (49.5%) were males, and 534 (50.5%) were females. Of the study participant, 827 (78.4%) were Kuwaiti, 224 (21.2%) were non-Kuwaiti Arab, and 4 (0.4%) were non-Kuwaiti non-Arabs. It was found that 374 (36.5%) participants were in the age range of 21-30, and 394 (38.4%) were 31-40 years old. On enquiring about the monthly income in Kuwaiti Dinars (KD), 471 (44.6%) participants were earning 500-1000 KD, 286 (27.1%) had 1000-1500 KD, 68 (6.4%) had 1501-2000 KD, and 36 (3.4%) ranged of 2001 KD or more. Study participants were enrolled from various ministries, which included Commerce and Industry (n=153, 14.5%), Defense (n=133, 12.6%), Finance (n=181, 17.1%), Foreign Affairs (n=125, 11.8%), Interior (n=38, 3.6%), Education (n=176, 16.7%), Health (n=149, 14.1%), and Communication (n=102, 9.6%) (Figure 1, Table 2). Regarding years of work experience, 359 (34.2%) were between 1-4 years, 415 (39.5%) were between 5-14 years, and 227 (26.4%) were between 15 or more years of employment. The distribution of variables, including weekly working hours, perception of home, work environment, and sleep quality, are given in Table 3.

**Table 2:** Socio-demographic characteristics of the participants enrolled in our study n: Number, %: Percentage

Characteristics	n	(%)
Years of work		
1-4 years	359	34.2
5-14 years	415	39.5
15 years or more	277	26.4
Time of work		
At the morning	1030	97.6
At the evening	10	0.9
Shift based	15	1.4
Hours of work per week		
30 hours or less	282	26.8
30-35	369	35.1
35-40	225	21.4
40-45	101	9.6
45 or more	75	7.1
Description of work as stressful		
Strongly disagree	124	11.8
Disagree	241	22.9
Neutral	418	39.7
Agree	207	19.6
Strongly agree	64	6.1
Sleep disturbed by work		
Strongly disagree	159	15.1
Disagree	288	27.3
Neutral	254	24.1
Agree	262	24.8
Strongly agree	92	8.7
Description of home as stressful		
Strongly disagree	381	36.2
Disagree	319	30.3
Neutral	242	23
Agree	82	7.8
Strongly agree	29	2.8

**Table 3:** Socio-demographic characteristics of the participants n: Number, %: Percentage KD: Kuwaiti Dinars (The official currency of Kuwait)

Characteristics	n	(%)
Gender		
Male	523	49.5
Female	534	50.5
Age		
21-30	374	36.5
31-40	394	38.4
41-50	195	19
51-65	63	6.1
Nationality		
Kuwaiti	827	78.4
Non-Kuwaiti Arab	224	21.2
Non-Kuwaiti Non- Arab	4	0.4
Governorate		
Al-Ahmadi	64	6.1
Capital	273	25.8
Al-Farwaniyah	253	23.9
Hawalli	293	27.7
Al-Jahrah	63	6
Mubarak AlKabeer	111	10.5
Marital status		
Single	364	34.4
Married	630	59.6
Divorced	54	5.1
Widow	9	0.9
Income (KD/month)		
500 or less	194	18.4
500-1000	471	44.6
1000-1500	286	27.1
1500-2000	68	6.4
2000 or more	36	3.4
Ministry		
Commerce	153	14.5
Communication	75	7.1
Education	176	16.7
Finance	181	17.1
Health	149	14.1
Interior	38	3.6
Defense	133	12.6
Foreign	125	11.8
Others	27	2.6

Distribution of tobacco products used among workers in various ministries

Of 1057 participants, 275 (26%) were smokers of at least one product. Among smokers, 16 (1.5%) were using three tobacco products, 62 (5.9%) used two tobacco products, and 197(18.6%) reportedly used only one tobacco product. Of the smokers, 214 (20.3%) were exclusively cigarette smokers, and 46 (21.8%) of them started at the age of 15 years or less. Regarding the daily dose of cigarette smoking, reportedly 81 (38%) smoked less than one pack, 94 (44.1%) smoked one pack, 33 (15.5%) smoked two packs, and 5 (2.3%) more than two packs. Of the smokers, 94 (8.9%) participants used shisha, and 74 (82.2%) started at 25 years or less. Of the smokers, 61 (5.8%) smoke e-cigarettes, and 40 (66.8%) of them start at 30 years or younger. The frequency of using shisha and e-cigarettes among the participants is shown in Table 4.

**Table 4:** The distribution of tobacco products-use practices among the participants n: Number, %: Percentage KD: Kuwaiti Dinars (The official currency of Kuwait)

Smoking practice	n	(%)
Smoking (any product)		
Yes	275	26.0
No	782	74.0
Concurrent use of multiple tobacco products		
Three	16	1.5
Two	62	5.9
One	197	18.6
None	782	74.0
Cigarette smoking		
No	841	79.7
Yes	214	20.3
Age at onset of cigarette smoking		
15 or less	46	21.8
16-20	119	56.4
21 or more	46	21.8
Number of packs per day		
Less than one pack	81	38.0
One pack	94	44.1
Two packs	33	15.5
More than two packs	5	2.3
Shisha smoking		
No	962	91.1
Yes	94	8.9
Age at onset of shisha smoking		
20 or less	27	30.0
21-25	47	52.2
26 or more	16	17.8
How often smoke shisha a week		
Once	30	31.9
Twice	22	23.4
Three times	10	10.6
More than three times	32	34.0
E-cigarette smoking		
No	994	94.2
Yes	61	5.8
Age at onset of E-cigarette smoking		
25 or less	17	28.8
26-30	23	38.0
31 or more	19	32.2

Order of tobacco product used at the onset of smoking

**Figure 2:**
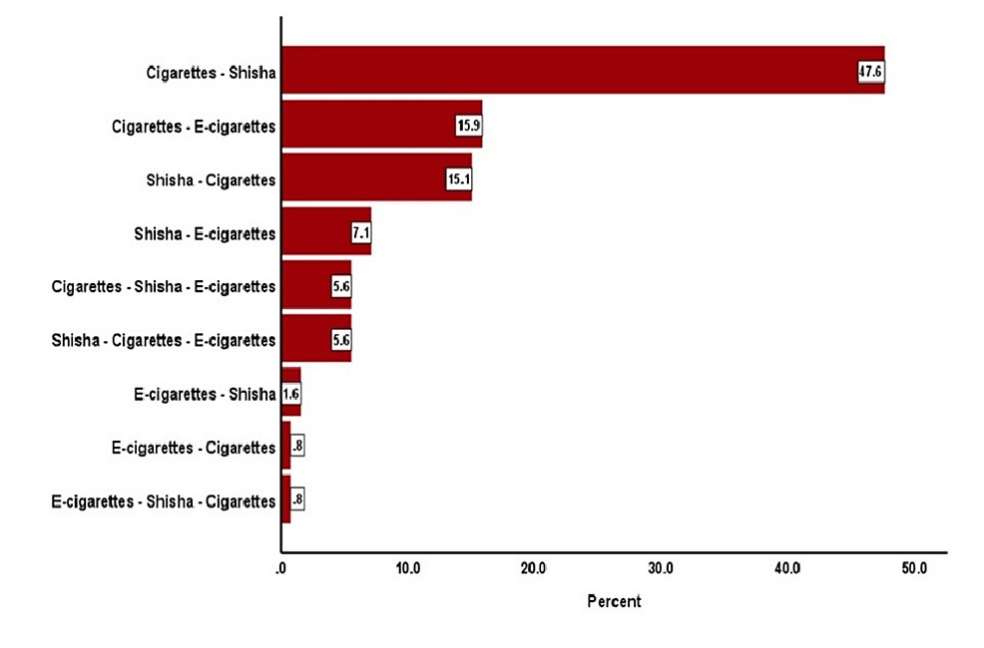
Order of indulging into different tobacco products by smokers among the participants

Figure 2 shows the order of indulging in using tobacco products among smokers. Most started smoking cigarettes, followed by shisha (47.6%). Among the smokers, 15.9% started with cigarettes and then moved to e-cigarettes. Nearly an equal proportion (15.1%) of smokers first began with shisha smoking before they indulged in cigarette smoking. Some other orders of a multiplicity of tobacco products used by smokers in this study are also shown in Figure 2.

Chi-square analysis of the association between socio-demographics, comorbidities, and smoking status

The socio-demographic variables that were significantly associated with smoking of at least one product included: gender (p< 0.001), nationality (p= 0.049), total monthly income in KD (p= 0.003), and ministry of employment (p< 0.001). Age, marital status, time of work, hours of work per week, description of work as stressful, disturbed by work, description of home as stressful, heart disease, high blood pressure, diabetes, obesity, upper respiratory tract infection, peptic ulcer disease, and sleep disturbances were not significantly associated with smoking of at least one product (Table 5, 6).

**Table 5:** Chi-square analysis of the association between smoking status and socio-demographic variables measured on the participants n: Number, %: Percentage KD: Kuwaiti Dinars (The official currency of Kuwait)

Variables	Total (n)	Smokers n (%)	p-value
Gender			< 0.001
Male	523	243 (46.5)	
Female	534	32 (6.0)	
Age			0.795
21-30	374	97 (25.9)	
31-40	394	106 (26.9)	
41-50	195	46 (23.6)	
51-65	63	17 (27.0)	
Nationality			0.049
Kuwaiti	827	204 (24.7)	
Non-Kuwaiti Arab	228	71 (31.1)	
Marital status			0.627
Never married	364	98 (26.9)	
Ever married	693	177 (25.5)	
Income (KD/month)			0.003
500 or less	194	52 (26.8)	
501-1000	471	112 (23.8)	
1001-1500	286	69 (24.1)	
1501-2000	68	31 (45.6)	
>2000	36	11 (30.6)	
Ministry			< 0.001
Commerce	153	37 (24.2)	
Communication	102	30 (29.4)	
Education	176	35 (19.9)	
Finance	181	61 (33.7)	
Health	149	21 (14.1)	
Interior	38	23 (60.5)	
Defense	133	37 (27.8)	
Foreign	125	31 (24.8)	
Hours of work per week			0.512
30 hours or less	282	60 (21.3)	
31-35	369	104 (28.2)	
36-40	225	66 (29.3)	
41-45	101	28 (27.7)	
46 hours or more	75	15 (20.0)	

**Table 6:** Chi-square analysis of the association between smoking status and socio-demographic variables measured on the participants n: Number, %: Percentage

Variables	Total (n)	Smokers n (%)	p-value
Description of work as stressful			0.546
Strongly Disagree	124	31 (25.0)	
Disagree	241	62 (25.7)	
Neutral	418	120 (28.7)	
Agree	207	48 (23.2)	
Strongly Agree	64	14 (21.9)	
Sleep disturbed by work			0.721
Strongly Disagree	159	42 (26.4)	
Disagree	288	81 (28.1)	
Neutral	254	69 (27.2)	
Agree	262	61 (23.3)	
Strongly Agree	92	22 (23.9)	
Description of home as stressful			0.400
Strongly Disagree	381	86 (22.6)	
Disagree	319	86 (27.0)	
Neutral	242	70 (28.9)	
Agree	82	24 (29.3)	
Strongly Agree	29	8 (27.6)	
Heart disease			0.667
No	1025	269 (26.2)	
Yes	28	6 (21.4)	
Hypertension			0.056
No	941	236 (25.1)	
Yes	116	39 (33.6)	
Diabetes			0.578
No	985	254 (25.8)	
Yes	72	21 (29.2)	
Obesity			1.000
No	796	207 (26.0)	
Yes	261	68 (26.1)	
Upper respiratory tract infection			0.445
No	932	238 (25.5)	

Multivariable binary logistic regression model of factors associated with the smoking practice of any product

Male compared to female workers had statistically significantly (p< 0.001) higher odds of being a smoker of at least one tobacco product (adjusted OR= 15.3, 95% CI= 10.0-23.4). Participants were significantly (p= 0.009) more likely to use at least one tobacco product if their monthly income ranged from 501-1000 KD (adjusted OR= 1.9, 95% CI= 1.2-3.0) or 1501-2000 KD (adjusted OR= 2.3, 95% CI= 1.2-4.5) compared to those who had monthly income range 500 KD or less. Compared to the workers of the Ministry of Commerce and Industry, the Ministry of Interior's workers had statistically significantly (p= 0.009) higher odds of being a smoker of at least one tobacco product (adjusted OR= 3.9, 95% CI= 1.6-9.4) (Table 7).

**Table 7:** Binary logistic regression of significant factors associated with a multiplicity of tobacco product use among the participants OR: odds ratio,  CI: confidence interval KD: Kuwaiti Dinars (The official currency of Kuwait) Ref: Reference for comparison

Characteristics	Crude OR	95% CI		Adjusted OR	95% CI	p-value
Gender						
Female	1.00	Ref		1.00	Ref	<0.001
Male	13.62	9.15-20.24		15.32	10.03-23.39	
Income (KD/month)						
500 or less	1.00	Ref		1.00	Ref	0.009
500-1000	0.85	0.58-1.24		1.92	1.22-3.01	
1000-1500	0.87	0.57-1.31		1.29	0.80-2.08	
1500-2000	2.29	1.29-4.05		2.30	1.17-4.54	
2000 or more	1.20	0.55-2.61		0.78	0.32-1.90	
Ministries						
Commerce and Industry	1.00	Ref		1.00	Ref	0.009
Defense	1.21	0.71-2.05		0.80	0.44-1.48	
Finance	1.6	0.98-2.58		0.93	0.52-1.63	
Foreign affairs	1.03	0.59-1.79		0.87	0.46-1.65	
Interior	4.88	2.27-10.16		3.83	1.57-9.39	
Education	0.79	0.46-1.31		0.69	0.38-1.26	
Health	0.52	0.28-0.92		0.62	0.31-1.22	
Communication	1.31	0.74-2.29		1.31	0.66-2.61	

## Discussion

Prevalence of tobacco smoking

Our study assessed the prevalence and factors associated with the use of tobacco (cigarette, shisha, and e-cigarette) in both genders, 21 years old or older, public-sector workers of eight of 14 ministries (Commerce and Industry, Defense, Finance, Foreign Affairs, Interior, Education, Health, and Communication) of Kuwait. In our study, the prevalence of current smoking was higher among males (46.5%) than females (6.0%) - evidence of substantial increase over the previously reported corresponding figures of (34.4%) and (1.9%) among males and females, respectively, in Kuwait [9]. Most of the smokers in this study were older than 51 years (27.0%), which overlaps with the results of current smoking in the United States as it was highest in the young adult population [11]. In our study, the prevalence of concurrent use of three, two, or one tobacco products was 1.5%, 5.9%, and 18.6%, respectively. Of the participants, 20.3% were exclusive cigarette smokers. Although tobacco consumption including cigarette smoking has been declining since the mid-1960s, cigarettes remain by far the most commonly used tobacco product as reported in the United States [11]. This is also associated with a report in China, whose people smoke more than 40% of all cigarettes globally [12]. Despite anti-tobacco consumption, legislation was done and promulgated from time to time, yet tobacco consumption through different modalities of tobacco use continues to be high in Kuwait. Therefore, mass education through print and electronic media could reduce tobacco consumption in Kuwait. On the other hand, the results showed that the percentages of using two and three tobacco products were relatively low (5.9% and 1.5%, respectively). In addition, 8.9% of the workers were shisha smokers, and 5.8% were e-cigarette smokers. In our study, the percentage of shisha smokers was almost the same as reported by Gaza strip refugee students and in Lebanon. However, the proportion of shisha smokers in our study (94 participants, 8.9%) was much smaller than that reported from Syria (30%) [13]. People in the Middle East are using shisha as a cultural tradition, and the onset of smoking is strongly influenced by similar practices by peers and families [14]. The prevalence of using e-cigarettes in our study was almost similar to that reported in New England, New York, and New Jersey. The main reason cited by e-cigarette users in the United States was that e-cigarettes help in smoking cessation [15]. However, this aspect needs further investigation.

Multivariate logistic regression

A significant and independent association existed between males and using one or more tobacco products. These results agree with the findings of a previous study, which reported that males were more likely to use tobacco products in Kuwait [9].

In addition, the high income of the participants was a strong and significant predictor of using one or more tobacco products. Thus, participants with higher incomes, presumably, are more willing to buy and indulge in using one or multiple tobacco products. This result is congruent with a previous study, which found that people with relaxed budgets are more flexible in spending on purchasing tobacco products compared to those with a low income [16]. According to reports from Gambia, individuals with low income and limited wealth were more likely to develop a smoking habit. The prevalence of smoking among this demographic group may be attributed to the stressful nature of their lives, which may lead to the use of smoking as a coping mechanism. This observation highlights the potential impact of socioeconomic factors on health behaviors and underscores the need for targeted interventions to address the root causes of health disparities [17]. However, another study in the United Kingdom showed an inverse relationship between high income and smoking tobacco products. They concluded that people with high incomes are more educated than those with lower incomes, and they recognized the adverse effects of smoking on their health [18]. 

Changing in smoking behaviors/modalities 

The present study showed that smokers usually shift between different tobacco products. This change in smoking behavior may be due to trying to quit smoking (i.e., shifting from cigarettes to e-cigarettes) or only to change the taste (i.e., trying different flavors of e-cigarettes). Either way will probably lead to acquiring the habit of using more than one tobacco product. Interestingly, one participant reported that he smokes e-cigarettes secretly during official working hours (indoors), cigarettes with tea and coffee, and shisha when gathering with friends. In the present study, most smokers (47.6%) began with cigarettes and then shifted to shisha. This percentage is similar in the Gulf countries, especially Saudi Arabia [19].

Similarly, in Iran, most university students still believe that shisha is the healthiest choice among tobacco products and is one of their traditional forms [20]. Others were going to shisha smoking, preferring different flavors like fruits, candy, and chocolates. For the same reason, shisha smoking is practiced by women in the United States [21]. According to the findings in our study, a portion of the individuals who were observed smoking cigarettes initially (15.9%) subsequently transitioned to using e-cigarettes. Conversely, it was observed that a subset of the participants (15.1%) initiated their tobacco use with shisha smoking, but later transitioned to cigarette smoking. The shift from using cigarettes to e-cigarettes was less frequent among the final-year pharmacy students in Serbia (9.9%) than in our present results. 

Limitations and Recommendations

Some of the limitations of this study should be noted while interpreting the results. First, smoking status was self-reported; therefore, the true prevalence of smoking in Kuwait may be somewhat underestimated, particularly among females. This opinion may be attributed to cultural norms that dictate that smoking is morally unacceptable for women. As such, females may experience societal pressure to report their smoking status due to the perception of shame associated with this behavior. Second, the low prevalence of self-reported tobacco smoking among females precluded the stratified analysis for males and females to examine the gender-specific risk factors. Therefore, recall or information bias might have occurred as females who were smokers might be reluctant. This aspect warrants attention in future research to ensure the questionnaire's confidentiality, for instance, by incorporating anonymous data collection, private interviews, and non-judgmental language. Some examples of confidential questionnaires could be using coded identifiers instead of personal information, such as name or address, to maintain anonymity or providing the option for participants to complete the questionnaire online or through mail to avoid face-to-face interviews that might discourage candid responses.

Another limitation of our study is that the cause-effect relationship cannot be drawn from the observed associations as the nature of this study is cross-sectional. In addition, our study found that individuals with high incomes had a higher likelihood of smoking. However, the specific type of tobacco product preferred by this group was not identified, as well as their costs. Additionally, there needs to be more knowledgeable concerning the tobacco product preferences of low-income employees. Lower-income individuals may prefer cigarettes over other types due to their lower cost. Therefore, future studies should aim to determine the preferred type of tobacco product for each group depending on their income better to understand the relationship between income and tobacco product preference. We also recommend that future studies incorporate nicotine, tar, and carbon monoxide measurements for each tobacco product to identify which types pose more significant health risks. Elevated levels of these substances can result in severe illnesses. Finally, future research endeavors in this field should consider the limitations of our study while also considering the recommendations presented above. Doing so will promote a more profound comprehension of smoking behavior across diverse socioeconomic strata.

## Conclusions

A higher prevalence of smoking among males (46.5%) and females (6.4%) was recorded than previously reported estimates in Kuwait. Among the participants, 18.6% used one tobacco product exclusively, while 7.4% used two or three. The high income of the participants was a significant predictor of the use of one or more multiple tobacco products. Anti-smoking campaigns through mass education using electronic and print media may help reduce the prevalence of tobacco smoking. Increasing taxes on tobacco products may discourage the purchase and use of tobacco products in this study population, particularly in low-income individuals. 
